# Marsupial chromosome DNA content and genome size assessed from flow karyotypes: invariable low autosomal GC content

**DOI:** 10.1098/rsos.171539

**Published:** 2018-08-29

**Authors:** Fumio Kasai, Patricia C. M. O'Brien, Jorge C. Pereira, Malcolm A. Ferguson-Smith

**Affiliations:** Department of Veterinary Medicine, University of Cambridge, Cambridge, UK

**Keywords:** marsupial genome, chromosome profile, mammalian evolution

## Abstract

Extensive chromosome homologies revealed by cross-species chromosome painting between marsupials have suggested a high level of genome conservation during evolution. Surprisingly, it has been reported that marsupial genome sizes vary by more than 1.2 Gb between species. We have shown previously that individual chromosome sizes and GC content can be measured in flow karyotypes, and have applied this method to compare four marsupial species. Chromosome sizes and GC content were calculated for the grey short-tailed opossum (2*n* = 18), tammar wallaby (2*n* = 16), Tasmanian devil (2*n* = 14) and fat-tailed dunnart (2*n* = 14), resulting in genome sizes of 3.41, 3.31, 3.17 and 3.25 Gb, respectively. The findings under the same conditions allow a comparison between the four species, indicating that the genomes of these four species are 1–8% larger than human. We show that marsupial genomes are characterized by a low GC content invariable between autosomes and distinct from the higher GC content of the marsupial × chromosome.

## 1. Introduction

Marsupials are unique among mammals for their small diploid number of chromosomes ranging from 10 to 24, with one known exception, *Aepyprymnus rufescens*, which has 32 [1]. It has been proposed that the putative ancestral marsupial karyotype has a diploid number of 14 [1,2], whereas the putative ancestral eutherian karyotype has a diploid number of 44 or 46 [3,4]. Reciprocal chromosome painting between Australian and South American marsupials confirms the remarkable chromosome conservation among phylogenetically diverged groups [1,2,5]. Although cross-species chromosome painting using human probes works in eutherian mammals including Afrotheria and Xenarthra (except for the Y chromosome) [3,6], only the human X chromosome paint probe has been successful on marsupial chromosomes [7]. This has been explained by the divergence time between marsupials and eutherians, estimated to be approximately 147.7 Myr [8]. As marsupials comprise one of three major lineages in mammals, analysis of marsupial genomes could allow further elucidation of ancestral mammalian genomes.

Total amounts of DNA and their GC content are fundamental parameters characterizing each genome. An early study demonstrated marsupial genome size estimates for 13 species, ranging from 3.4 to 4.6 Gb [9]. Another study reported that analysis of 32 samples from different experiments including different methods gave marsupial genome sizes ranging from 2.8 to 5.5 Gb with a median of 4.1 Gb [10]. The wallaby genome size was estimated to be 2.9 Gb from the average of results from three different methods which give variable values between 2.46 and 3.74 Gb [11]. Because these discrepancies could not be explained by variation within the species, they are assumed to be due to methodological problems in measuring genome sizes, which could lead to different estimates for the same species [12]. This is partly because tissue samples include cells at various stages of the cell cycle and because staining of nuclear DNA is influenced by accessibility of dye molecules [12]. Previous data obtained under different conditions could cause considerable misinterpretation when used for comparisons between species.

Theoretically, genome size and GC content can be determined at the highest resolution and at the base pair level by whole-genome sequencing. However, the total number of nucleotides has not yet been determined for any vertebrate, including opossum [13], wallaby [11] and Tasmanian devil [14] as it has been found that gaps and errors occur during the assembly of sequence reads due to difficulties with the inclusion of repeats [15]. This is most apparent in the well-characterized latest human genome reference, GRCh38, which still includes gaps accounting for at least 151 Mb, corresponding to more than 4.9% of the whole genome [16]. Therefore, the GRCh38 data which show 3029 Mb can be regarded as an estimate.

Chromosomes are identified by size and GC content in flow karyotypes [17–20] and the size of each human chromosome has been estimated by quantitative analysis [21]. Our previous studies show that flow karyotypes are reproducible and demonstrate that chromosome size and GC content can be estimated in flow karyotypes using human chromosomes as a reference [19,20,22]. Although the results cannot be expected to provide the actual DNA content, the relative chromosome size and GC content in each sample allow comparisons between chromosomes in each genome and between genomes of different species.

It is known that coding sequences have a relatively high GC content [23] and that GC content is highly correlated with the recombination rate [24]. Transmission of GC-alleles over AT-alleles increases GC content, associated with GC-biased gene conversion [25]. This leads to the formation of GC-rich segments in regions with high levels of recombination [26]. A recombination-driven increase in GC content has been reported in primates [24] and mice [26]. Because the total genome GC content varies between species [27], its analysis is important for our understanding of genome evolution particularly in marsupials in which genome content appears to be different from eutherians.

In this study, we measured chromosome sizes and GC content in four marsupial species from their flow karyotypes. The largest chromosome in each species accounts for approximately 20% of the genome and our estimations of the genome sizes are close to human. The marsupial chromosome profiles show little variation in GC content between autosomes and a high GC content in the X chromosome, distinct from human. These features distinguish the marsupial chromosomes from other mammals.

## 2. Material and methods

Chromosome preparations for flow karyotyping were made according to conventional protocols [28]. Briefly, fibroblast cultures were made from a fat-tailed dunnart (*Sminthopsis crassicaudata*, SCR), a tammar wallaby (*Macropus eugenii*, MEU), a grey short-tailed opossum (*Monodelphis domestica*, MDO) and Tasmanian devil (*Sarcophilus harrisii*, SHA), as used in our previous studies [5,29,30]. An EBV-transformed normal male human lymphoblastoid cell line was used to provide a reference flow karyotype. Mitotic cells were collected after colcemid treatment (KaryoMAX^®^ Colcemid™, Gibco) and treated with hypotonic solution. The cell pellet was resuspended in ice-cold polyamine buffer and incubated on ice for 10 min. The suspension was then vortexed vigorously for 12 s and the chromosomes were stained by 5 µg ml^−1^ of Hoechst 33258 (Sigma-Aldrich, B2883), 40 µg ml^−1^ of chromomycin A3 (Sigma-Aldrich, C2659) and 10 mM MgSO_4_. Before the analysis, 10 mM sodium citrate and 25 mM sodium sulfite were added.

For measurement, each chromosome sample was mixed with the human reference sample and run on a flow cytometer, MoFlo (Beckman-Coulter) equipped with two water-cooled lasers, consisting of multiline UV at 330–360 nm and light at 457 nm which excite Hoechst 33258 and chromomycin A3, respectively. Each chromosome position in flow karyotypes was measured using Adobe Photoshop. The chromosome size was calculated as the relative distance between the origin and the projection of the peak for each chromosome on the line through the origin and human chromosome 4 (HSA-4) using Microsoft Excel. These values were converted into absolute DNA size using a reference size of 200 Mb for HSA-4 on each flow karyotype [21]. This was calibrated by a separate calculation based on the peak positions of HSA-17 (89 Mb), confirmed by the size of HSA-19 (66 Mb). These reference sizes are estimates obtained in the previous study using the same method of flow karyotyping [21]. The GC content of each chromosome was calculated from the ratio obtained from the AT and GC fluorescence values on the flow karyotype using the reference GC content of HSA-4 (38.2%), HSA-17 (45.5%) and HSA-19 (48.4%) [31]. The identity of chromosomes in the flow karyotypes was based on previous studies (electronic supplementary material, figure S1) [5,29,30].

## 3. Results

**Figure 1.**
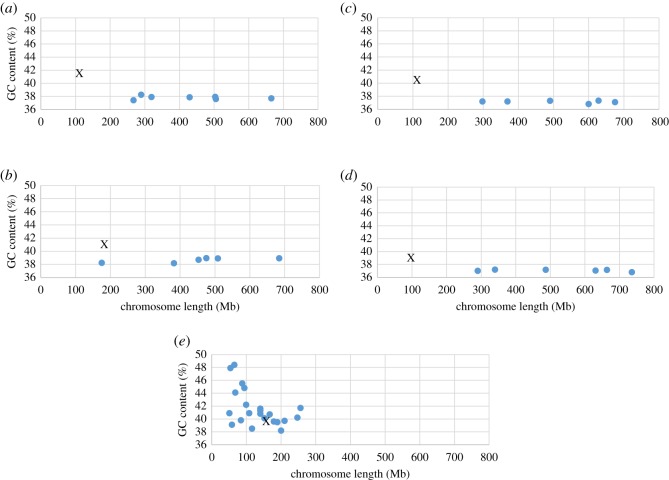
Chromosome profiles based on flow karyotype measurements showing the relationship between chromosome size and GC content in the grey short-tailed opossum (2*n* = 18) (*a*), tammar wallaby (2*n* = 16) (*b*), Tasmanian devil (2*n* = 14) (*c*) and fat-tailed dunnart (2*n* = 14) (*d*) compared with human (2*n* = 46) (*e*) at the same scale. X chromosomes are indicated by X in each profile. Marsupial autosomes are variable in size, but have an invariable GC content. By contrast, marsupial X chromosomes have the highest GC content in each genome.

**Figure 2.**
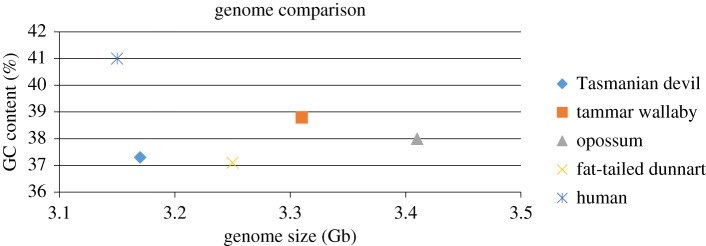
Comparison of total genome size and GC content between four marsupials and human. Marsupial genome sizes are larger than those of human in excess of 10%. Low GC content in marsupials can be distinguished from the comparatively high GC content in human.

The nine MDO, eight MEU, seven SHA and seven SCR chromosomes resolved into individual peaks in the flow karyotypes, except for MEU 4 and 5 that are similar in size and GC content and so sort together in the same peak (electronic supplementary material, figures S2 and S3). The size and GC content of each chromosome were determined from the flow karyotypes and are shown in table 1. Chromosome profiles for each species based on size and GC content are shown in figure 1. The chromosome sizes broadly range between 102 and 736 Mb, with an average of 379 Mb for MDO, 414 Mb for MEU, 453 Mb for SHA and 464 Mb for SCR. The largest chromosome in each species is between 665 and 736 Mb, representing between 19.5 and 22.7% of the genome and the smallest autosome is MEU 7 accounting for 5.2% of its genome. The largest chromosome among these four species is SCR 1 estimated to be 736 Mb, accounting for 22.7% of its genome. This is much more than the sum of human chromosomes 1 and 2 accounting for 8.1% and 7.9% of the human genome, respectively, which together amount to approximately 500 Mb. A comparison of flow karyotypes between the Tasmanian devil and opossum in the mixed sample demonstrates that MDO 1, 2 and 5 are larger than their homologous chromosomes SHA 2, 4 and 6 respectively, and that MDO 8 is smaller than SHA 5 [14]. These differences are consistent with our quantitative data and support our measurements. Genome size based on the sum of each chromosome size revealed 3408 Mb for MDO, 3314 Mb for MEU, 3173 Mb for SHA and 3250 Mb for SCR. These are all larger than human, but the difference is less than 8% of the human genome (figure 2).

**Table 1.** Size and GC content of each chromosome and each total genome (TG).

Chr	opossum (MDO)			Wallaby (MEU)			Tasmanian devil (SHA)			Dunnart (SCR)		
	size (Mb)	proportion (%)	GC (%)	size (Mb)	proportion (%)	GC (%)	size (Mb)	proportion (%)	GC (%)	size (Mb)	proportion (%)	GC (%)
1	665	19.5	37.7	684	20.6	38.9	675	21.3	37.1	736	22.7	36.8
2	503	14.7	37.9	508	15.3	38.9	628	19.8	37.3	664	20.4	37.2
3	505	14.8	37.5	475	14.3	38.9	600	18.9	36.8	631	19.4	37.1
4	429	12.6	37.9	453	13.7	38.7	490	15.4	37.3	487	15.0	37.2
5	319	9.4	37.9	453	13.7	38.7	369	11.6	37.1	340	10.5	37.2
6	289	8.5	38.2	382	11.5	38.2	298	9.4	37.2	290	8.9	37.2
7	267	7.8	37.4	175	5.3	38.2						
8	319	9.4	37.9									
X	112	3.3	41.3	184	5.6	40.8	113	3.6	40.2	102	3.1	39.0
TG	3408	100	37.9	3314	100	38.8	3173	100	37.3	3250	100	37.1

The range of chromosome GC content, with the exception of the X chromosome, is similar in each species and between 36.8% and 38.9%. The X chromosomes have the highest GC content in each genome (39–41%). It is reported that all opossum chromosomes in the flow karyotype are shifted towards the chromomycin axis compared to devil chromosomes [14], and this is consistent with our measurements of GC content. Similar genome size and total GC content of SHA and SCR are reflected by their close phylogenetic relationship.

## 4. Discussion

We demonstrate that the genome sizes measured from our flow karyotypes of opossum (3.41 Gb) and Tasmanian devil (3.17 Gb) are in good agreement with their measurements of 3.48 and 3.17 Gb, respectively, from genome sequencing data [13,14]. In contrast to these two well-organized sequencing analyses, the wallaby assembly, Meug_2.0, consists of 324,751 scaffolds with an N50 of 34.3 kb, providing an estimate for the genome size of 3.66 Gb [11]. Because of the large number of scaffolds, the wallaby genome sequencing data have not reached the level needed for the estimation of genome size and show a considerable difference from our measurement of 3.3 Gb. High consistency between the two measurements by whole-genome sequencing and flow karyotyping in opossum and Tasmanian devil suggests that our results from flow karyotyping provide good estimates of genome sizes in the wallaby and fat-tailed dunnart.

Our results on devil chromosome size are not consistent with the previous data [14]. It appears, from the methods described in that study, that the DNA line used for the calculation does not fit correctly in the flow karyotype because the position of the origin is misplaced. This caused an error in constructing the parameters of the flow karyotype and the different conditions have led to different results for DNA content. Because of this, the autosome size above the reference chromosome size is larger in our study than in the previous data and the X chromosome below the reference marker is smaller in our study than in the previous data. Our results on genome sizes in the four marsupial species measured from flow karyotypes are between 3.17 and 3.41 Gb, i.e. 1–8% larger than that in human (3.15 Gb; figure 2). The similarities in genome sizes with less than 10% variation between species could be related to their high level of chromosome conservation revealed by cross-species chromosome painting [1,5], implying that large-scale gross changes have not occurred in marsupial genomes during their evolution.

Although marsupial chromosomes have a wide distribution in size in each species, our analyses reveal that the GC content is similar in both large and small autosomes (figure 1). This feature can be observed in marsupial flow karyotypes [5,29,30], in which the chromosomal peaks are aligned diagonally indicating their similarity in chromosomal AT : GC ratios (electronic supplementary material, figures S1–S3). However, previous measurements of genome size from flow karyotypes in opossum and wallaby based on chromosome size describe considerable variations in chromosome GC content between 34% and 42%, and between 27% and 37%, respectively [11]. These data do not fit with the distribution of peaks in their flow karyotypes, and this factor must be responsible for the miscalculation of DNA content for each peak and the inconsistencies in chromosome size in their report. Our results on GC content now provide good correspondence with the flow karyotypes and genome sequencing data [13,14] and correct previous measurements in opossum and wallaby [11]. The autosomes of mammals including humans have chromosomes that vary in GC content [20], and this is different from marsupials, which demonstrate low variation in autosomal GC content. The same low variation pattern is observed in macro-chromosomes of reptiles and birds, although their micro-chromosomes have a higher GC content [19].

It is postulated that the gene content of the X chromosome is highly conserved among mammals because of the strong selection needed to maintain dosage compensation [32]. The marsupial X chromosome is homologous to the long arm and pericentric region of the short arm of the human X chromosome [7] and eutherian mammals have autosomal additions to the X chromosome since the time of their divergence from marsupials [33]. The human X chromosome has a relatively low GC content (39%) compared with the genome average of 41%. However, the GC content of the X chromosome in marsupials is significantly higher than the autosomal GC content. This accumulation is supposed to have happened in the marsupial genomes since their divergence from eutherian mammals [34].

A comparison of mean GC content at the third-codon position (GC3) among mammals demonstrates that the opossum is the GC-poorest with 43.89% [27]. The estimated ancestral GC3 of the eutherian is 46.16%, equivalent to the human GC3 [23]. It has also been suggested that the putative ancestral eutherian genome structure is close to the human pattern [35]. Although no general decline in the GC content has been observed during eutherian evolution, the GC content is decreased in marsupial genes [27,36]. The variable model suggests that the opossum genome lacks GC-rich isochore structures [37], and that the sequence composition is relatively homogeneous compared to human, mouse, dog and chicken [13].

Chromosomal regions of high GC are unusually susceptible to breakage and consequent rearrangement [38]. In the human karyotype, GC poor chromosomes tend to be large and experience less recombination [38]. Most marsupials have small chromosome numbers and, as expected, larger chromosomes are found in those with fewer chromosomes. It is noted that recombination rate is negatively correlated with chromosome size [24]. The low GC content in marsupial autosomes may be associated with reduced rates of evolutionary rearrangement [33]. Stability of the opossum karyotype suggests that most of the genome has experienced low recombination rates over an extended period [13]. Because the GC content can be increased by the process of GC-biased gene conversion [39], a lower GC content in marsupial autosomes throughout evolution could be due mainly to a reduction in recombination rate and a reduced rate of gene conversion [36]. These features are in good agreement with the limited number of evolutionary chromosome rearrangements identified among diverse marsupials [5].

Although low GC content in the total genome can be explained by these mechanisms, this does not entirely explain the unique feature of invariable GC content between autosomes. If the putative ancestral therian genome had a variable autosomal GC content, the rates of recombination would have to have been highly controlled to evolve the invariable GC content of marsupial autosomes. On the other hand, the chromosome GC content observed in the putative ancestral marsupial karyotype might have been conserved in modern marsupials and could have been present also in the therian common ancestor.

## Supplementary Material

Supplementary figures 1, 2, 3

## Data accessibility

Flow karyotypes used for the measurements are given in the electronic supplementary material.
