# Supplementary material for: Marsupial chromosome DNA content and genome size assessed from flow karyotypes: invariable low autosomal GC content
Source: R Soc Open Sci. 2018 Aug 29;5(8):171539. doi: 10.1098/rsos.171539 (PMC6124049; doi:10.1098/rsos.171539)
Supplement: Supplementary figures 1, 2, 3 [file rsos171539supp1.pdf]

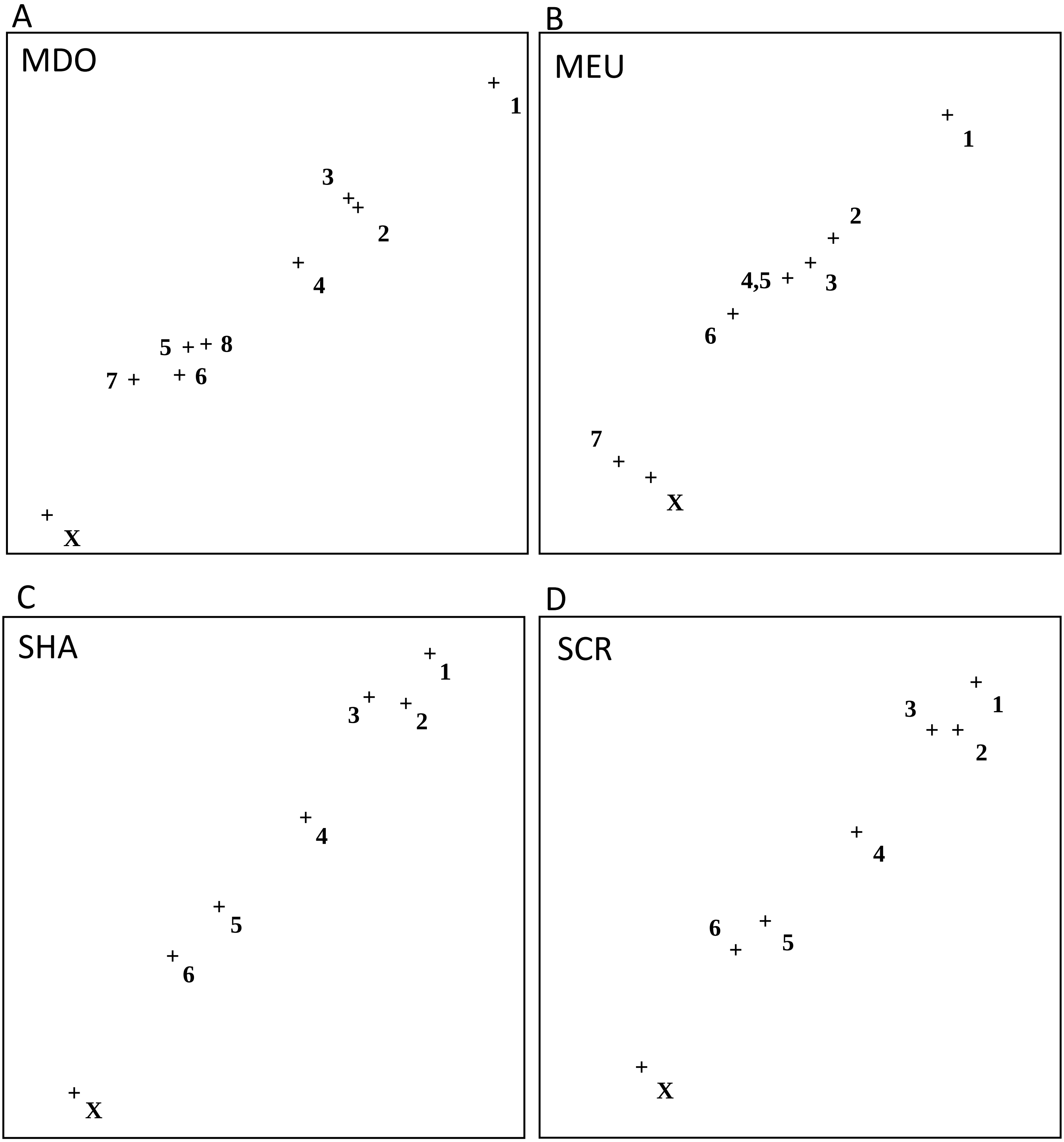

Supplementary figure 1.
Diagrams illustrating the relative peak positions of chromosomes and their assignment from previous studies.（A) Gray short-tailed opossum, MDO (2n=18), (B) tammar wallaby, MEU (2n=16), (C) Tasmanian devil, SHA (2n=14) and (D) fat-tailed dunnart, SCR (2n=14) [5,28,29]. The peak positions are applied in the present study as a means of identification of the chromosome peaks used in the calculation of chromosome size and GC content.

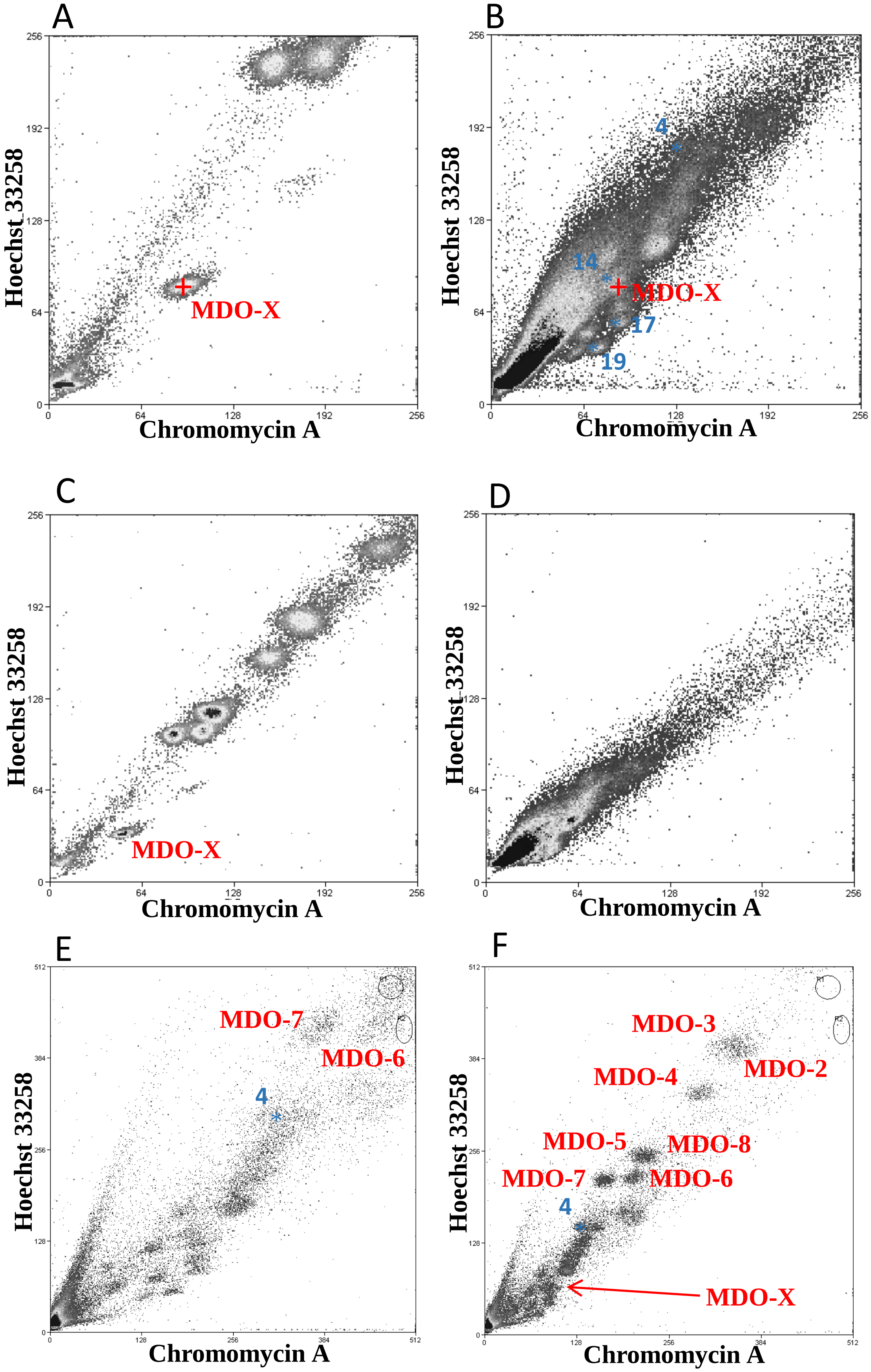

Supplementary figure 2.
Opossum partial (A) and whole (C) flow karyotypes. Under the same sorting conditions, the opossum chromosome sample was replaced by the human reference sample and sorted separately. Opossum flow karyotypes A and C correspond to human B and D, respectively. Peak positions of human chromosomes 4, 17 and 19 (blue numbers)used for references are indicated in B. The mixed sample was also sorted and peaks of both opossum and human appear in the same karyotypes (E and F). The peak position of opossum X chromosome is indicated by MDO-X, showing that the size and GC content are close to human chromosomes 14.

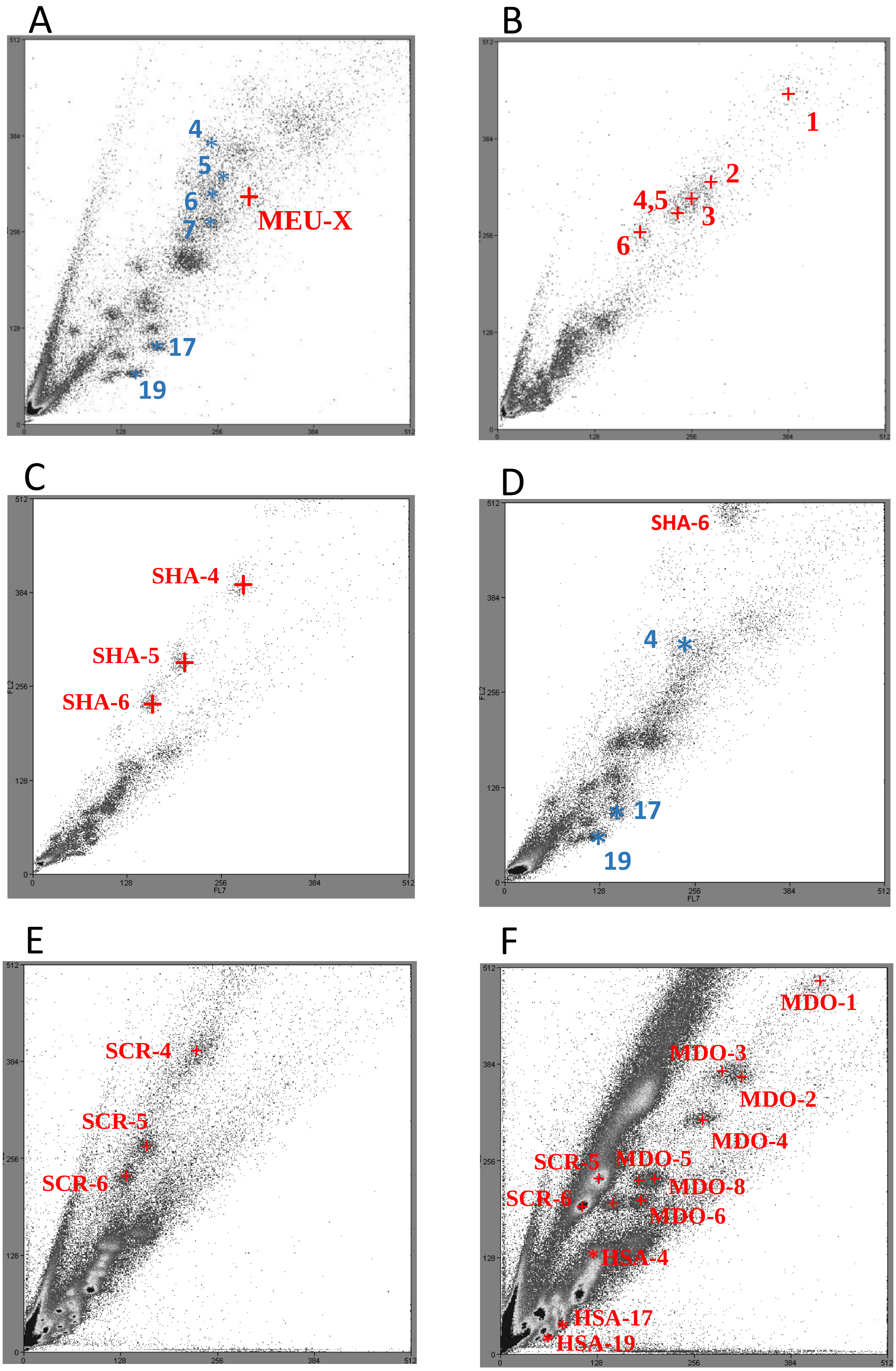

Supplementary figure 3.
The mixed sample of wallaby and human was sorted and both peaks appear in the same karyotypes (A and B). Peak positions of human chromosomes 4, 5, 6, 17 and 19 are indicated in A and D. The peak position of wallaby X chromosome is indicated by MEU-X, showing that the size is similar to human chromosomes 5 and 6, and that the GC content is close to human chromosome 7. The partial Tasmanian devil flow karyotype is shown in a mixed sample with human under different magnification (C and D). The partial Sminthopsis flow karyotype is shown in a mixed sample with human (E). Three samples, dunnart, opossum and human, were mixed together and the flow karyotype shows a merged image of the three species. This clearly shows that the GC content of dunnart chromosomes is lower than in opossum.
